# Integrated analysis of mRNA and microRNA expression profiles in hepatopancreas of *Litopenaeus vannamei* under acute exposure to MC-LR

**DOI:** 10.3389/fgene.2023.1088191

**Published:** 2023-01-19

**Authors:** Dajuan Zhang, Lanying Huang, Yingxuan Jia, Shulin Zhang, Xiandong Bi, Wei Dai

**Affiliations:** Key Laboratory of Aquatic-Ecology and Aquaculture of Tianjin, College of Fishery, Tianjin Agricultural University, Tianjin, China

**Keywords:** *Litopenaeus vannamei*, MC-LR, hepatopancreas, RNA-seq, miRNA-seq, endoplasmic reticulum

## Abstract

Intensive shrimp farming is often threatened by microcystins Hepatopancreas is the primary target organ of MCs in shrimp. To investigate the response of hepatopancreas to acute MC-LR exposure, the expression profiles of RNA-seq and miRNA-seq in the hepatopancreas of *L. vannamei* were determined, and data integration analysis was performed at 72 h after MC-LR injection. The expression of 5 DEGs and three DEMs were detected by Quantitative PCR (qPCR). The results showed that the cumulative mortality rate of shrimp in MC-LR treatment group was 41.1%. A total of 1229 differentially expressed genes (844 up- and 385 down-regulated) and 86 differentially expressed miRNAs (40 up- and 46 down-regulated) were identified after MC-LR exposure. Functional analysis indicated that DEGs is mainly involved in the oxidative activity process in molecular functional categories, and proteasome was the most enriched KEGG pathway for mRNAs profile. According to the functional annotation of target genes of DEMs, protein binding was the most important term in the GO category, and protein processing in endoplasmic reticulum (ER) was the most enriched KEGG pathway. The regulatory network of miRNAs and DEGs involved in the pathway related to protein degradation in endoplasmic reticulum was constructed, and miR-181-5p regulated many genes in this pathway. The results of qPCR showed that there were significant differences in the expression of five DEGs and three DEMs, which might play an important role in the toxicity and hepatopancreas detoxification of MC-LR in shrimp. The results revealed that MC-LR exposure affected the degradation pathway of misfolded protein in ER of *L. vannamei* hepatopancreas, and miR-181-5p might play an important role in the effect of MC-LR on the degradation pathway of misfolded protein.

## Introduction

*Litopenaeus vannamei* is one of the most important economically farmed shrimp species in the world, providing a high-quality protein source for the human diet. Intensive shrimp farming has been developed for decades to meet the growing world market demand. However, intensive shrimp farming is generally accompanied by nutrient enrichment and gradual eutrophication (Yang et al., 2018), leading to the proliferation of algae, especially cyanobacteria (Alonso-Rodríguez and Páez-Osuna, 2003). Cyanobacteria can produce cyanotoxins, and microcystins (MCs), a group of hepatotoxins, are the most common cyanotoxins (Rastogi et al., 2014). The concentration of MCs in an aquatic environment could reach the levels as high as 2.5 mg/L (Sivonen and Jones, 1999). MCs exhibit high structural variability, and more than 100 variants of MCs have been identified (Li et al., 2021; Zhang et al., 2022), among which Microcystin-LR (MC-LR) is the most toxic and widely distributed (Puddick et al., 2014; Bouaïcha et al., 2019). MCs can accumulate in cultured shrimp, affect their growth and even lead to death directly (Merel et al., 2013).

The organic anion transporters expressed in liver/hepatopancreas have a high affinity for MCs (Zurawell et al., 2005), and liver/hepatopancreas is the most important target organ of MCs (Sebastián et al., 2015; Zhu et al., 2011). Many studies have demonstrated that MCs can inhibit the serine/threonine protein phosphatases by interacting with the catalytic subunits of PP1 and PP2A, and improve the activity of protein kinase (Zhou et al., 2015). High phosphorylation of various proteins in liver/hepatopancreas can lead to apoptosis through mitochondrial and endoplasmic reticulum pathways (Pavitt and Ron, 2012).

In the mitochondrial pathways, MCs can cause inhibition of protein phosphatases, change of reactive oxygen species content, antioxidant dysfunctions (Sebastián et al., 2015; Min et al., 2018; Zhang et al., 2019), enhancement of apoptosis-related enzyme activities, cell apoptosis (Chen et al., 2021; Sun et al., 2021), enhancement of apoptosis-related enzyme activities, cell apoptosis (Chen et al., 2021; Chi et al., 2021; ), and collapse of microfilaments, microtubules and intermediate filaments of the cytoskeleton (Zhou et al., 2015). MCs-induced accumulations of unfolded or misfolded proteins in the endoplasmic reticulum cavity leads to apoptosis through transcriptional induction of CHOP (Oyadomari and Mori, 2004) and/or caspase-12 dependent pathway (Szegezdi et al., 2003) in the endoplasmic reticulum pathways.

In recent years, transcriptome analysis has been performed to better understand the physiological functions and molecular biological processes of shrimp under environmental stress. Zhuo et al. (2021) found lysosome, sphingolipid metabolism and nitrogen metabolism related genes of *L. vannamei* were differentially expressed under cold stress. A total of 1024 genes were differentially expressed in *L. vannamei* fed with aflatoxin B1 (AFB1), being involved in functions, such as peroxidase metabolism, signal transduction, transcriptional control, apoptosis, proteolysis, endocytosis, and cell adhesion and cell junction (Zhao W. et al., 2017).

MicroRNAs (miRNAs) are short non-coding RNAs with a length of approximately 22 nucleotides that are involved in post-transcriptional regulation of gene expression in multicellular organisms (Kaufman et al., 2010). MiRNAs can degrade and/or repress the translation of target genes by binding to the specific sequences of mRNAs, usually located in the 3′ UTR (Bartel, 2004; Esteller, 2011). MiRNAs are involved in the regulation of shrimp and fish response to the environmental stress, such as pathogenic bacteria and heavy metals (Guo et al., 2018; Zheng et al., 2018; Zhao et al., 2020; Zhao et al., 2021). Under MCs stress, the expression profiles of miRNAs in *L. vannamei* have not been reported. In order to more clearly reflect the effects of MC-LR on the miRNAs and mRNAs in hepatopancreas of *L. vannamei*, acute toxicity test was performed in this study, and the expression profiles of miRNAs and mRNAs were analyzed by the next-generation sequencing technology. The results can provide valuable reference for revealing the molecular mechanism of hepatoxicity of MCs.

## Materials and methods

### Shrimp rearing and MC-LR preparation

*L. vannamei* (16.1 ± 1.0 g) were obtained from Haitong Jiangyang Aquaculture Professional Cooperative, Tianjin, China. Before being exposed to MC-LR, *L. vannamei* were acclimated to laboratory conditions in recirculation systems for 7 days, at optimal temperature of 27.5°C ± 1.0°C. Recirculation system consisted of three holding tanks (70 × 42 × 30 cm) and a filtering tank (70 × 42 × 50 cm). Each holding tank contained 80-L aerated tap water (pH about 7.0, dissolved oxygen about 7.0 mg/L, salinity about six) and covered with a plastic plate to prevent shrimp from jumping out of the tank. During acclimation, one-third of water was replaced daily. *L. vannamei* were fed once a day, with a feeding amount of 5 g per tank. The type of bait was the same as that used by shrimps in the breeding base. MC-LR (purity ≥95%) was purchased from Express Technology Co., Ltd. (Taiwan), and dissolved in the phosphate buffer before use.

### Toxicity test

Doses of MC-LR used in the experiment were based on the result from 72 h LD_50_ study of MC-LR in our pre-experiment of shrimp, which calculated the LD_50_ value (350 μg/kg BW) with a 95% confidence interval (264.50–553.52 μg/kg BW). Shrimps were randomly divided into two groups with three replicates per group and 30 shrimp per replicate. MC-LR solution and phosphate buffer (control group) were intramuscularly injected in the lateral area between the second and third abdominal segments of shrimp, respectively, with a dose of 20 μl using a 100 μl ml syringe. During the toxicity test, *L. vannamei* were not fed and the dead were removed out in time. When shrimp was poked with a glass stick, its inactivity was the standard of death.

### RNA extraction, strand-specific library construction and sequencing

After injection of MC-LR for 72 h, the hepatopancreas of ten shrimps in each replicated tank were collected, mixed, and stored at −80°C for RNA extraction. The hepatopancreas samples of control group (CK-1, CK-2, and CK-3) and MC-LR treatment group (T-1, T-2, and T-3) were delivered to the Guangzhou Gene *Denovo* Biotechnology Co., Ltd. (China) for total RNA isolation, library construction, and high throughput sequencing. Total RNA was extracted using Trizol reagent kit (Invitrogen, Carlsbad, CA, United States) according to the manufacturer’s protocol.

After total RNA was extracted, rRNAs and ncRNAs were removed to retain mRNAs. The enriched mRNAs were fragmented into short fragments by using fragmentation buffer and reverse transcribed into cDNA with random primers. Next, the cDNA fragments were purified with QiaQuick PCR extraction kit (Qiagen, Venlo, Netherlands), end repaired, poly(A) added, and ligated to Illumina sequencing adapters. Then UNG (Uracil-N-Glycosylase) was used to digest the second-strand cDNA. The digested products were size selected by agarose gel electrophoresis, PCR amplified, and sequenced using Illumina HiSeqTM 4000 (or other platforms).

After total RNA was extracted by Trizol reagent kit, the RNA molecules in a size range of 18–30 nt were enriched by polyacrylamide gel electrophoresis (PAGE). Then the 3′ adapters were added and the 36–48 nt RNAs were enriched. The 5’ adapters were then ligated to the RNAs as well. The ligation products were reverse transcribed by PCR amplification and the 140–160 bp size PCR products were enriched to generate a cDNA library and sequenced using Illumina HiSeq Xten by Gene *Denovo* Biotechnology Co. (Guangzhou, China).

### Bioinformatic analysis of mRNAs

Raw reads were further filtered by fastp (version 0.18.0) (Chen et al., 2018). Then, clean reads were mapped to the reference genome (NCBI accession number: ASM378908v1) using the HISAT2(version 2.1.0) (Kim et al., 2015).

Then String Tie was used to estimate expression values based on the alignment. mRNAs differential expression analysis was performed by DESeq2 (Li and Dewey, 2011) software between two different groups [and by edgeR (Love et al., 2014) between two samples]. The genes/transcripts with the parameter of false discovery rate (FDR) < 0.05 and absolute fold change≥2 were considered differentially expressed genes/transcripts. Differentially expressed mRNAs were subjected to enrichment analysis of GO functions (http://www.geneontology.org) and KEGG pathways (http://www.kegg.jp).

### Bioinformatic analysis of miRNAs

Removing adapters and low quality sequences of raw reads, the remaining reads (17–35 nt) were regarded as clean reads. All of the clean tags were aligned with small RNAs in GeneBank (Release 209.0) and Rfam(Release 11.0) database to identify and remove rRNA, scRNA, snoRNA, snRNA, and tRNA. All of the clean tags were then searched against miRBase database (Release 22) to identify known (Species studied) miRNAs (exist miRNAs).

The Miranda (Version 3.3a) and TargetScan (Version 7.0) were used to predict targets of the differentially expressed miRNAs. The default parameters of Miranda software are: the score threshold is 140, the energy threshold is −10 kcal/mol, and strict 5′seed pairing is required. The prediction method of TargetScan is to select the 2–8 nt sequence which start from 5′small RNA were choose as seed sequence to predict with 3′-UTR of transcripts. Then, the enrichment analysis of GO function and KEGG pathway was carried out for the target genes differentially expressing miRNAs.

### Validation of miRNA and mRNA profiles

To validate the accuracy of the result of differential expression, five DEGs and three differentially expressed miRNAs were selected for quantitative real-time PCR. Total RNA from the hepatopancreas of shrimp was isolated with Trizol reagent. mRNA and miRNA were reverse transcribed into cDNA by Hifair^®^ Ⅱ first Strand cDNA Synthesis Kit (Yeasen, China). The primers (Table 1) of mRNAs and miRNAs are designed by Guangzhou Regene Biotechnology Co. Ltd. (Guagnzhou, China). β-actin and U6 were used as an internal reference mRNAs and miRNAs, respectively. The qRT-PCR reaction system consists of 5 μl Hieff^®^ qPCR SYBR Green Master Mix (Low Rox Plus), 1 μl forward primer and 1 μl reverse primer, 2 μl cDNA and RNase-free Water to 10 μl. Transcript abundance of mRNA and miRNA were calculated using the comparative 2^−△△Ct^ method.

**TABLE 1:** mRNA and miRNA primers used for qRT-PCR validation.

Gene name	Accession number	Primer
eIF2a	ncbi_113822941	F: TGT​TGA​GCG​ATG​CAC​AGA​GA
		R: CCT​CAA​AGT​GCC​ATG​CAG​TC
Hspa4l	ncbi_113827731	F: TCC​GTA​GTG​GGG​AGG​AGA​AG
		R: ATG​CAG​AGG​AGT​CGA​TGC​AG
Ufd1	ncbi_113817522	F: ACC​AAC​CGA​CGT​GCC​AAT​AG
		R: CGT​AGC​AAC​AGG​GAG​TGA​CA
MAP3K5	ncbi_113816913	F: TGT​CAT​GGA​CCT​GGA​AGC​AG
		R: TCA​TGC​AGA​GGC​TGG​ACT​TC
let-70	ncbi_113829108	F: TAT​GGC​AGG​TGA​CGA​TGC​TG
		R: GGT​GAT​AAA​CTG​CCG​TGC​TG
β-actin	—	F: GCA​TCC​ACG​AGA​CCA​CCT​AC
		R: TTC​CTT​CTG​CAT​CCT​GTC​GG
U6	—	F: CTCGCTTCGGCAGCACA
		R: AAC​GCT​TCA​CGA​ATT​TGC​GT
miR-122-5P	—	GCC​GTG​GAG​TGT​GAC​AAT​GG
miR-222-3P	—	CGG​CAG​CTA​CAT​CTG​GCT​AC
miR-5119	—	CGGCGTCATCACATCCTG

## Results

### Cumulative mortality rate assay

**FIGURE 1:**
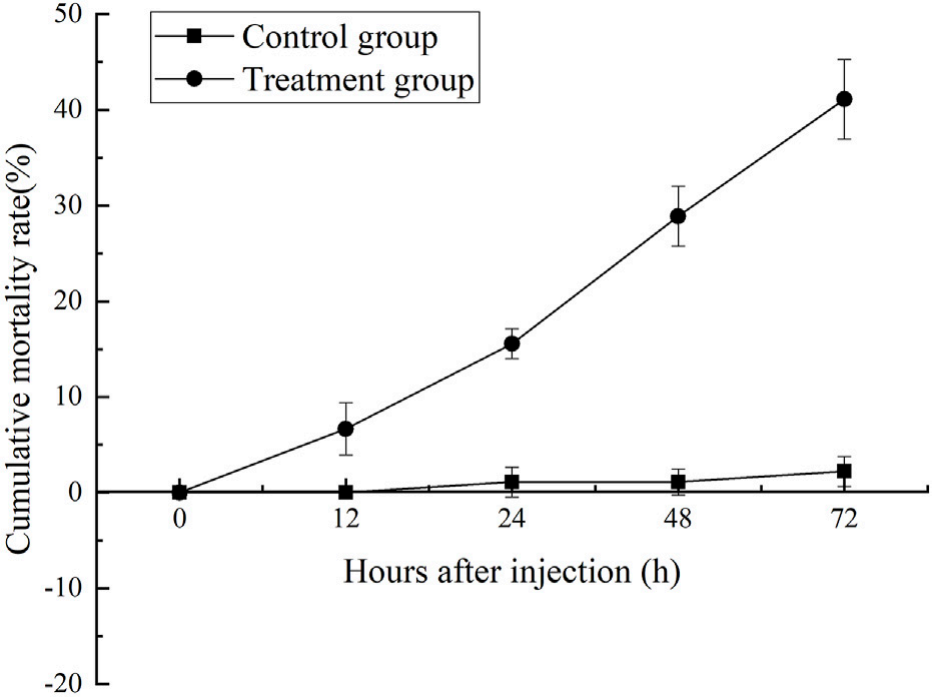
The cumulative mortality rate assay.

Changes in the cumulative mortality rate were shown in Figure 1. After MC-LR injection, the mortality rate of shrimp increased obviously with the extension of time after injection. After 12 h, the mortality of MC-LR injection group was significantly higher than that of control group (*p* < 0.05). After 72 h, the mortality rate in MC-LR treatment group was 41.1% and 2.2% in control group.

### mRNA sequencing and data analysis

Raw sequence data from six normalized libraries were concatenated and analyzed. The total number of raw reads ranged from 79 to 117 million. After quality control and filtering the raw reads, a total of 78–116 million clear readings were obtained, with Q30 > 93% and GC content >59%. About 85% of the effective reads were successfully mapped to *L. vannamei* genome. About 66% of the alignment was unique in the genome. Therefore, the differentially expressed genes (DEGs) analysis based on the genome was reliable.

### Differentially expressed transcripts analysis

**FIGURE 2:**
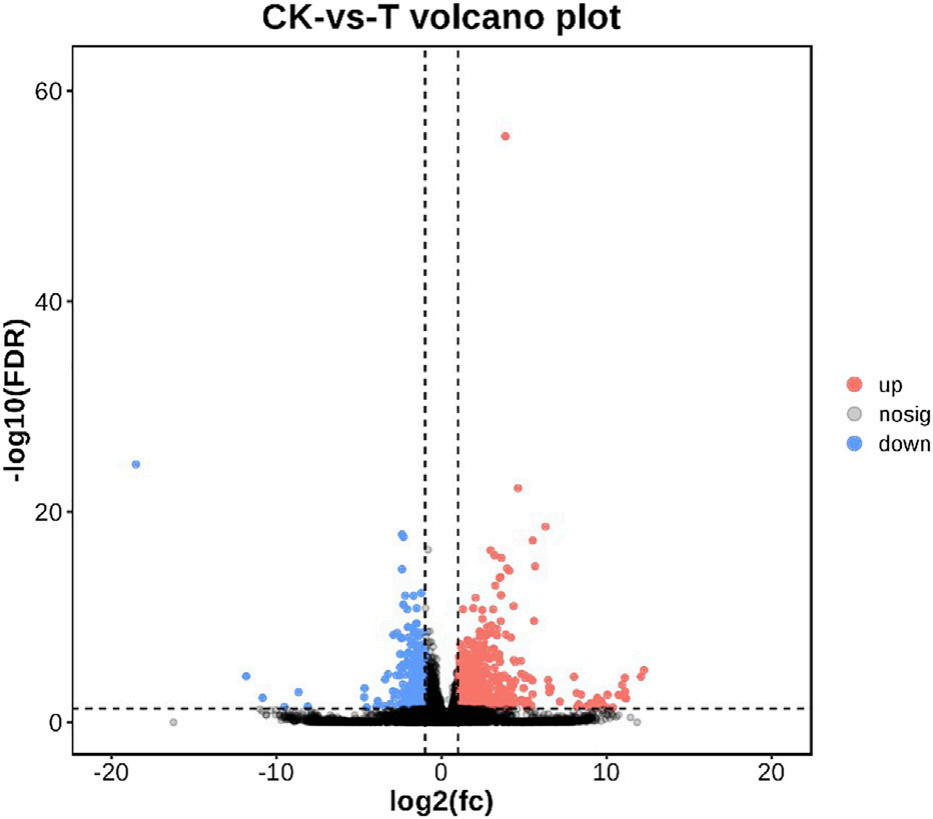
Volcano plot of differentially expressed genes. Up-regulated genes were showed as red dots, down-regulated genes were showed as blue dots, and no differentially expressed genes were shown as black dots.

As shown in Figure 2, a total of 18,462 genes were co-expressed by String Tie. After 72 h of injection, 1229 genes were differentially expressed in the control group and MC-LR treatment group. In MC-LR treatment group, 844 genes were significantly up-regulated and 385 genes were significantly down-regulated.

### GO and pathway enrichment analysis

**FIGURE 3:**
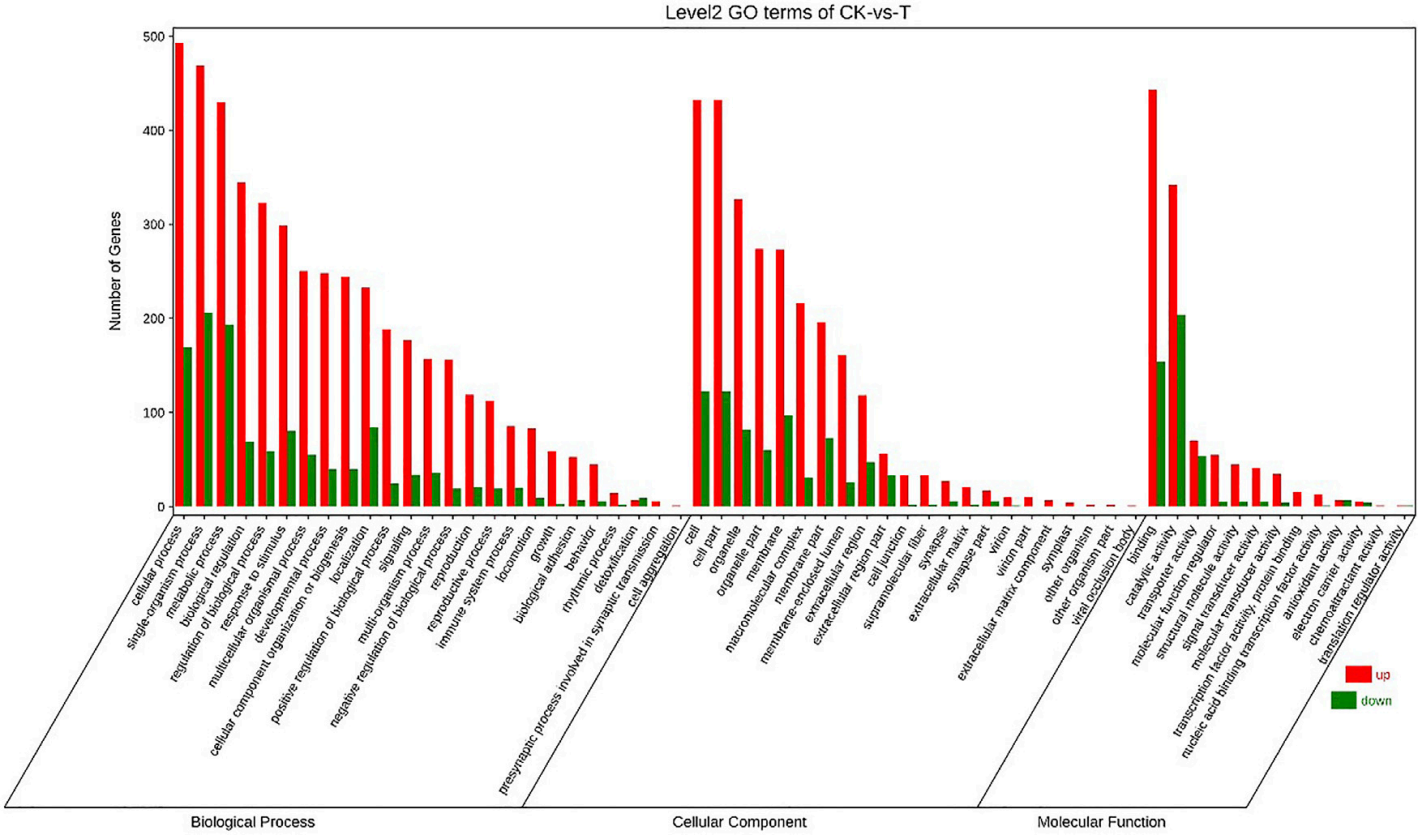
GO classification of genes.

Annotation of the DEGs was performed by GO. 25792 unigenes were annotated with GO, and a total of 17405 unigenes were successfully annotated to at least one GO term annotation. Based on the result of GO annotation, the genes were summarized into 60 groups belonging to three main categories (BP, Biological process; CC, Cellular component; MF, Molecular function). In the biological process, most genes were enriched in single-organism process, cellular process and metabolic process. In the cellular component, DEGs were mainly involved in the regulation of cell and cell part. In the molecular function, DEGs were mainly associated with binding and catalytic activity (Figure 3).

**FIGURE 4:**
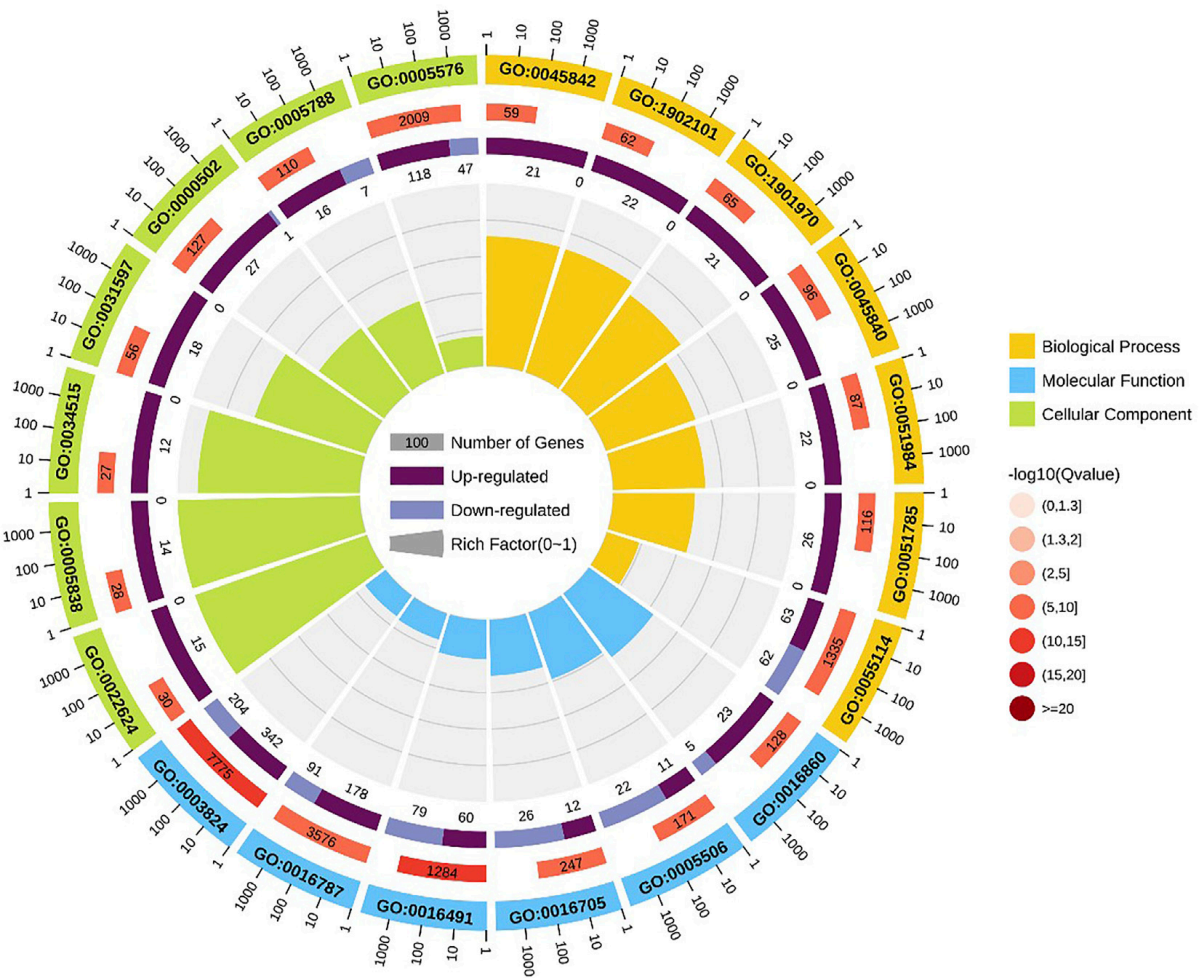
GO enrichment circle diagram of DEGs. The first circle: the top 20 GO term of enrichment, and the coordinate scale of the number of differential genes outside the circle. Different colors represent different Ontology; The second circle: the number and Q value of the GO term in the background of different genes. The more the number of different gene backgrounds, the longer the bar, the smaller the Q value and the redder the color. The third circle: bar chart of up-down differential gene proportion, deep purple represents up-down differential gene proportion and light purple represents down-down differential gene proportion; The specific values are displayed below; The fourth circle: Rich Factor value of each Gotham (the number of differential genes in this Gotham divided by all the numbers in this Gotham), background grid lines, each grid representing 0.1.

GO enrichment analysis indicated that positive regulation of metaphase/anaphase transition of cell cycle (GO: 1902101) was the most significant term in biological processes, followed by positive regulation of mitotic metaphase/anaphase transition (GO: 0045842) and positive regulation of mitotic sister chromatid separation (GO: 1901970). For cellular component, proteasome accessory complex (GO: 0022624) was the most significant term. For the category of molecular function, catalytic activity (GO: 0003824) and oxidoreductase activity (GO: 0016491) were the most significant term (Figure 4).

**FIGURE 5:**
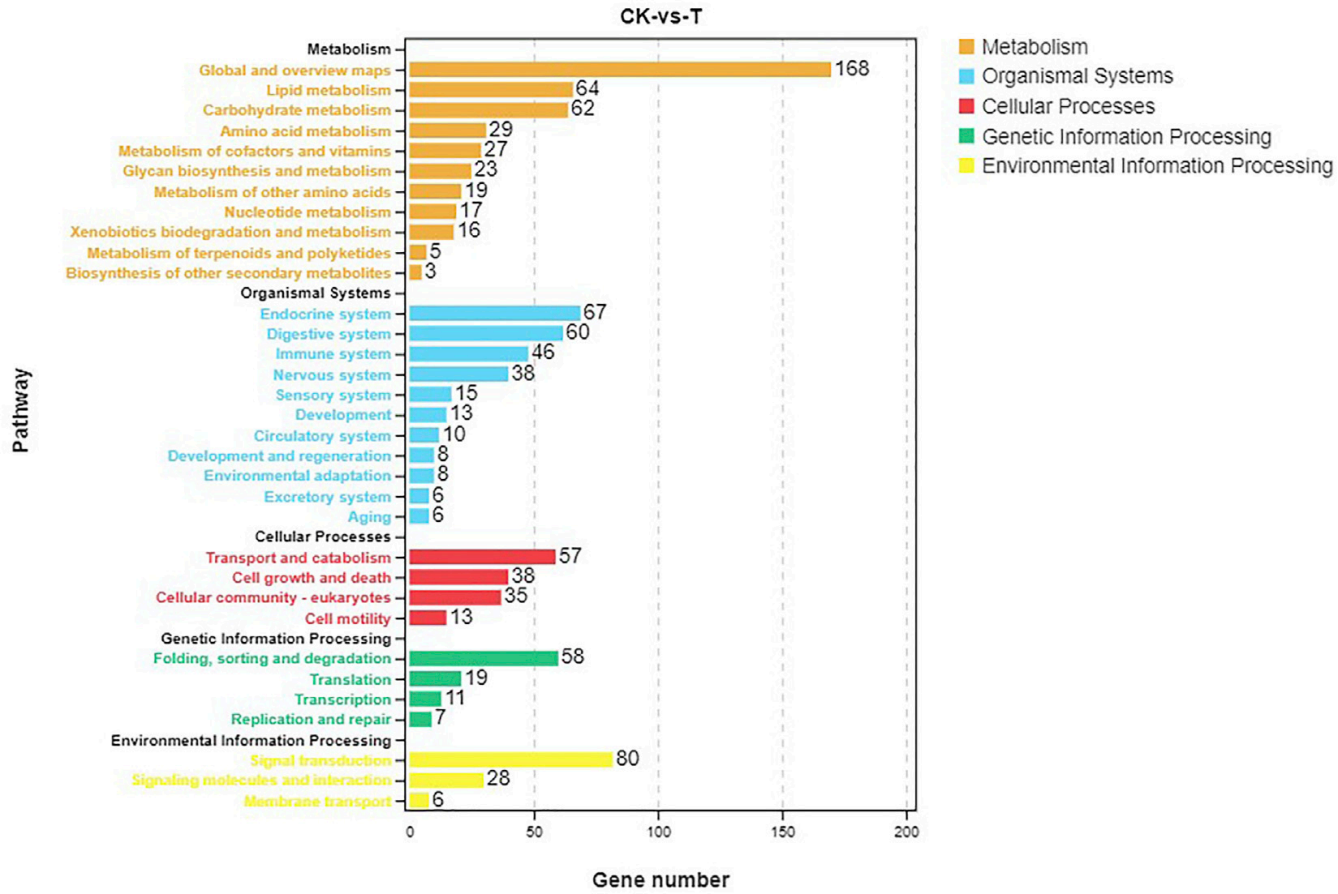
Pathway analysis based on KEGG.

**FIGURE 6:**
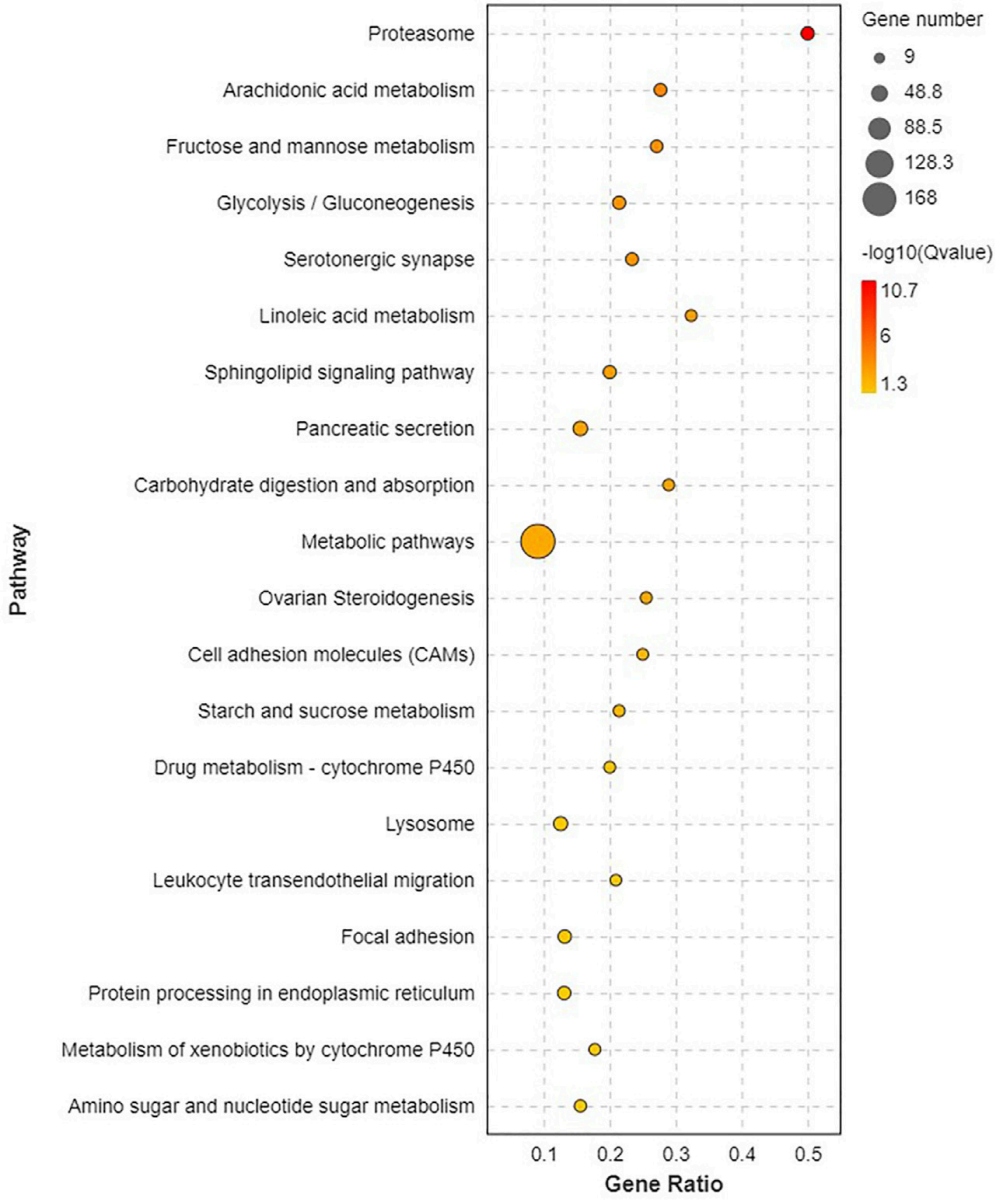
The first 20 KEGG pathway significantly enriched.

Pathway analysis was based on KEGG enrichment (Figure 5). A total of 1372 unigenes were divided into organismal systems, metabolism, cellular processes, environmental information processing and genetic information processing. Classification of KEGG pathway of DEGs is shown in Figure 6. Among them, proteasome (ko03050) was the most enriched pathway, followed by antifolate resistance (ko01523), linoleic acid metabolism (ko00591) and carbohydrate digestion and absorption (ko04973).

### miRNA sequencing and data analysis

MiRNAs libraries for the hepatopancreas samples from shrimps were sequenced. The total number of raw reads ranged from 15 to 24 million. After filtering the low-quality sequences, single-read sequences, and empty adaptors, a total of 14–22 million clean reads of 18–35 nt length were obtained. rRNA, tRNA, snRNA, and snoRNA were annotated based on GenBank and Rfam database.

### Identification of differentially expressed miRNAs and functional annotation of predicted target genes

**FIGURE 7:**
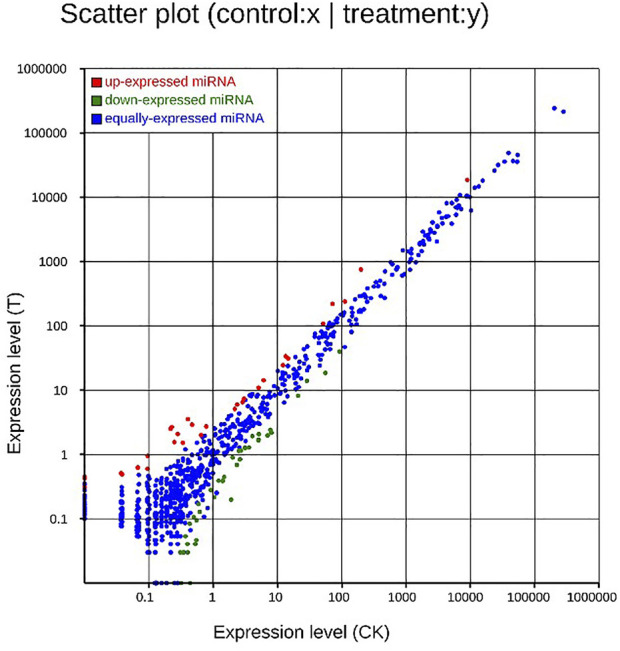
Scatter plot of DEMs. Up-regulated genes were showed as red dots, down-regulated genes were showed as green dots, and no differentially expressed genes were shown as blue dots.

**FIGURE 8:**
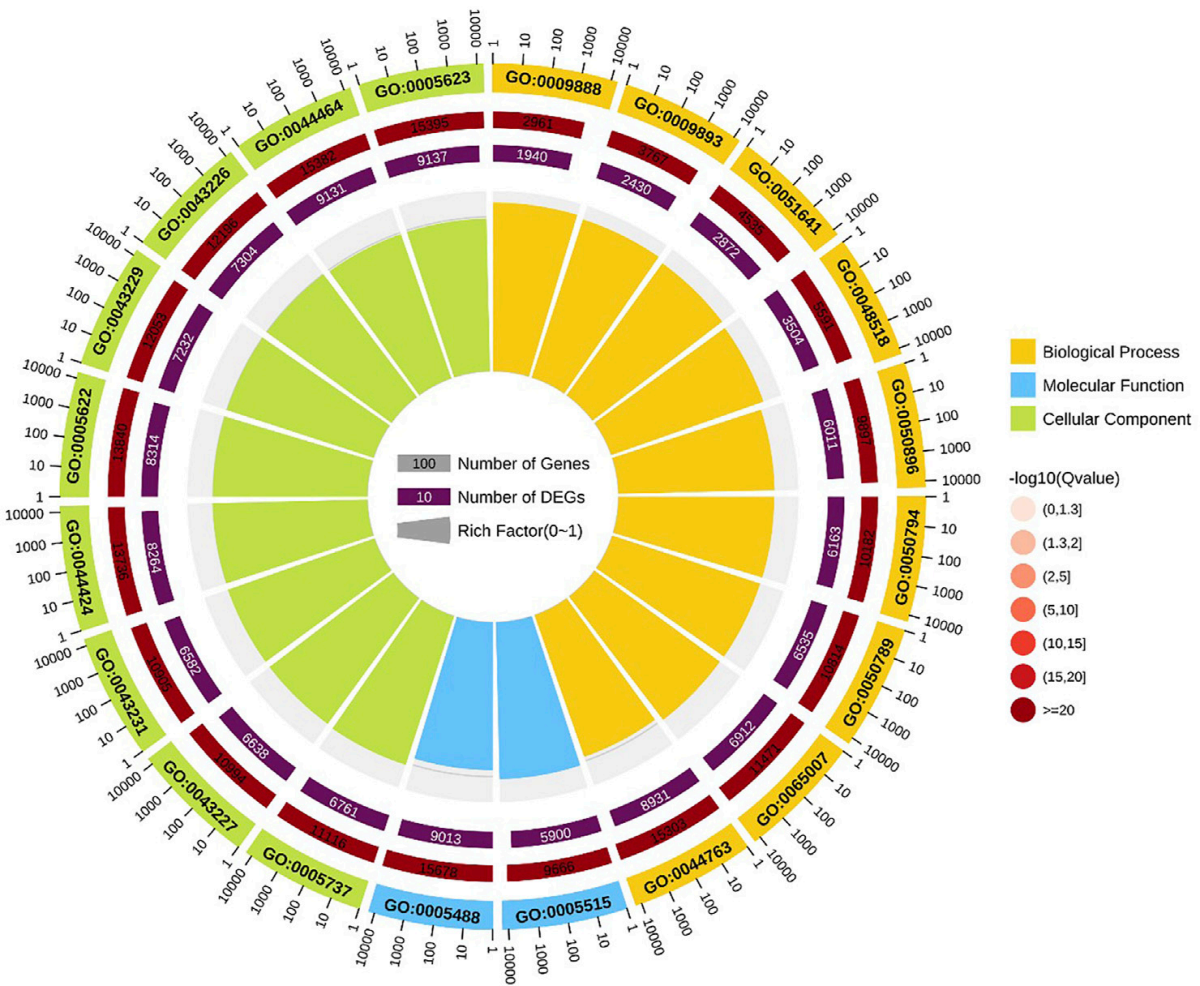
GO enrichment circle diagram of miRNAs target gene.

**FIGURE 9:**
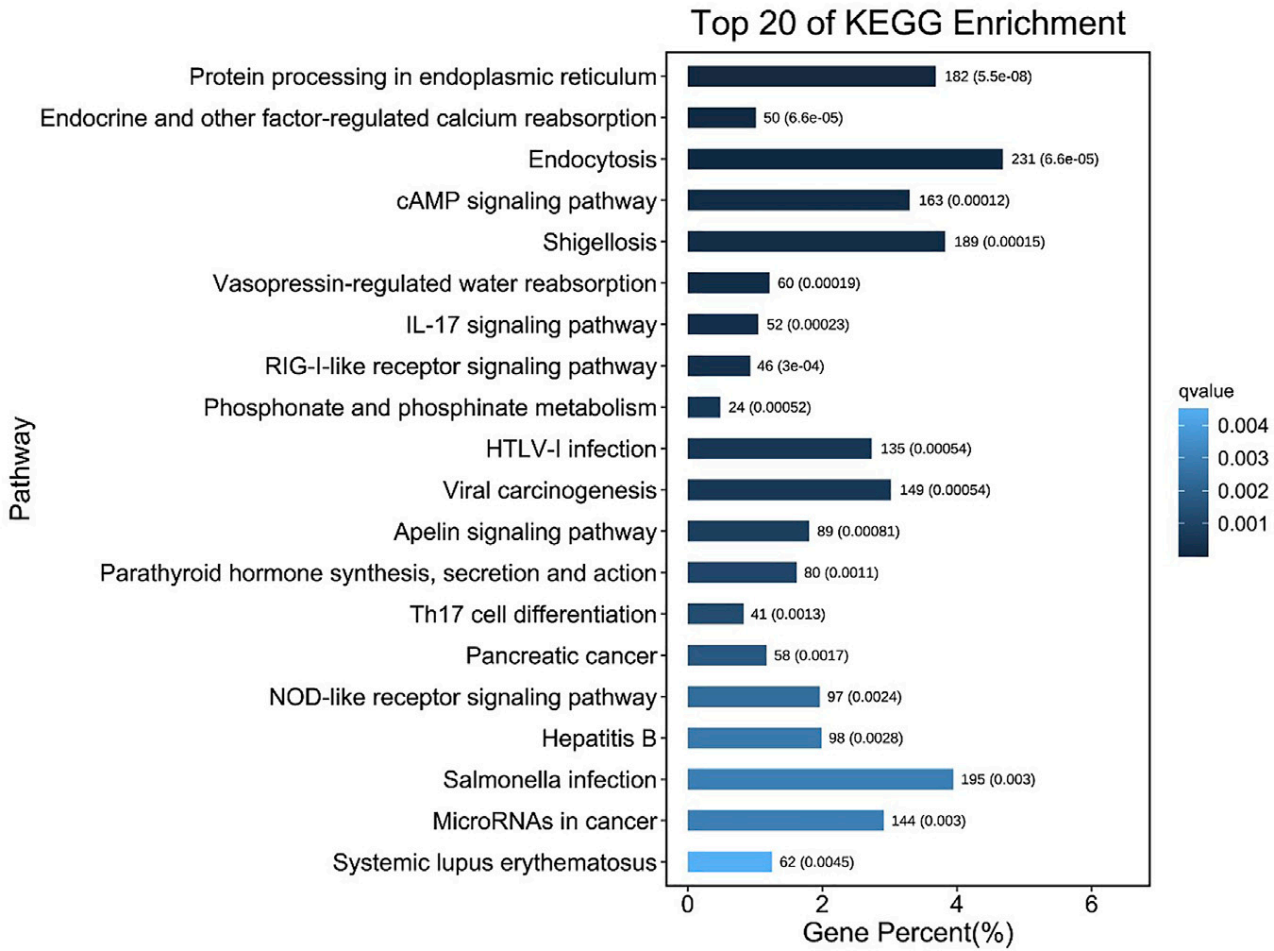
The top 20 pathways containing most number of DEmiRNAs target genes.

After MC-LR infection, differential expression of 907 miRNAs was analyzed. Among these miRNAs, 86 DEMs were identified, including 40 up-regulated and 46 down-regulated miRNAs (Figure 7). A total of 22,574 target genes were predicted by 907 miRNAs. GO enrichment analysis of DEMs predicted that the most significant cellular component term was cell part (GO:0044464), followed by cell (GO:0005623) and intracellular part (GO:0044424). For the category of molecular function, protein binding (GO:0005515) and binding (GO:0005488) was the most significant term. For biological process, biological regulation (GO:0065007) was the most significant term (Figure 8). KEGG pathway analysis displayed 348 pathways with the target genes were potentially regulated by the differentially expressed miRNAs. The results showed that protein processing in endoplasmic reticulum (ko04141) was the most enriched pathway followed by endocrine and other factor-regulated calcium reabsorption (ko04961), endocytosis (ko04144) and cAMP signaling pathway (ko04024) (Figure 9).

### Validation of RNA-seq and miRNA-seq data with qRT-PCR

**FIGURE 10:**
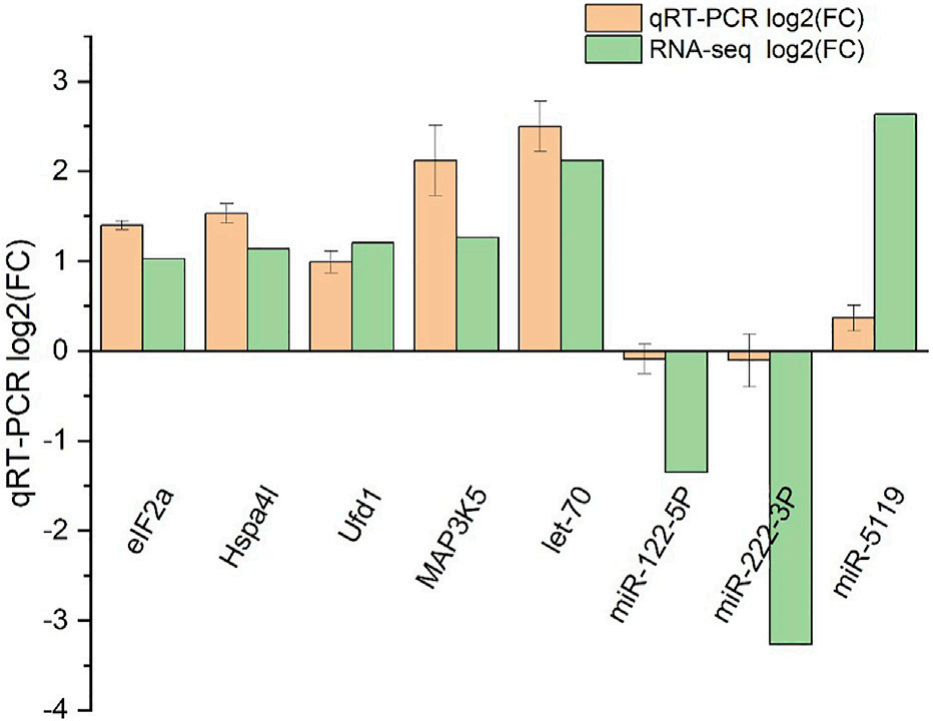
qRT-PCR validation of RNA-seq and miRNA-seq data. Negative values indicate the gene expression was down-regulated, while positive values indicate the gene expression was up-regulated.

Five DEGs and three DEMs identified from the sequencing data were analyzed for their expression using qRT-PCR (Figure 10). The expression pattern determined from the RNA-seq data was consistent with the expression pattern of selected genes determined using qRT-PCR, suggesting the RNA-Seq results were reliable.

### DEGs and DEMs involved in the degradation of misfolded protein in endoplasmic reticulum

MiRNA-mRNA interaction networks were constructed based on the 86 DEMs and 1229 DEGs, and a total of 1919 negative miRNA-mRNA pairs were associated. By the KEGG pathway analysis, a total of 280 pathways showed significant changes after acute exposure to MC-LR. Of the top 20 pathways, four genes were assigned to “Environmental information processing” and three to “organismal systems”. In addition, some important subclasses, including proteasome, Epstein-Barr virus infection, cell adhesion molecules (CAMs), shigellosis, carbohydrate digestion and absorption, small cell lung cancer, protein processing in endoplasmic reticulum, sphingolipid signaling pathway, toxoplasmosis, and starch and sucrose metabolism were also significantly enriched. These results suggested that the genes involved in these pathways might play a crucial role in the response of *L. vannamei* to MCs*.*

**FIGURE 11:**
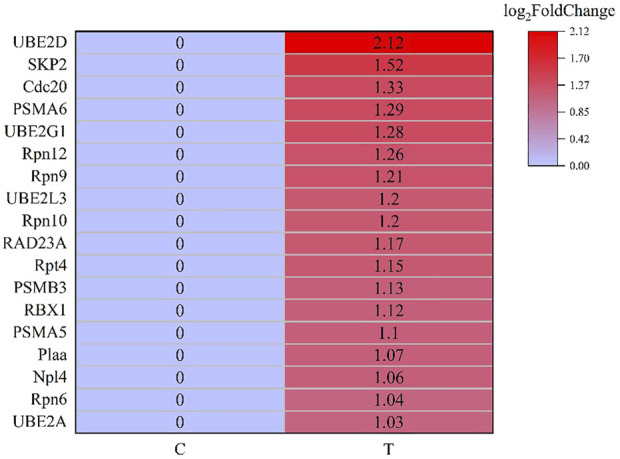
Heat map of DEGs involved in the degradation of misfolded protein in ER.

**FIGURE 12:**
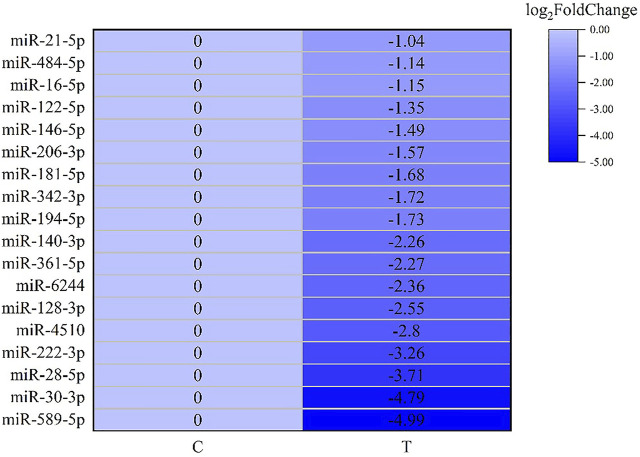
Heat map of predicted target genes of miRNAs involved in the degradation of misfolded protein in ER.

**FIGURE 13:**
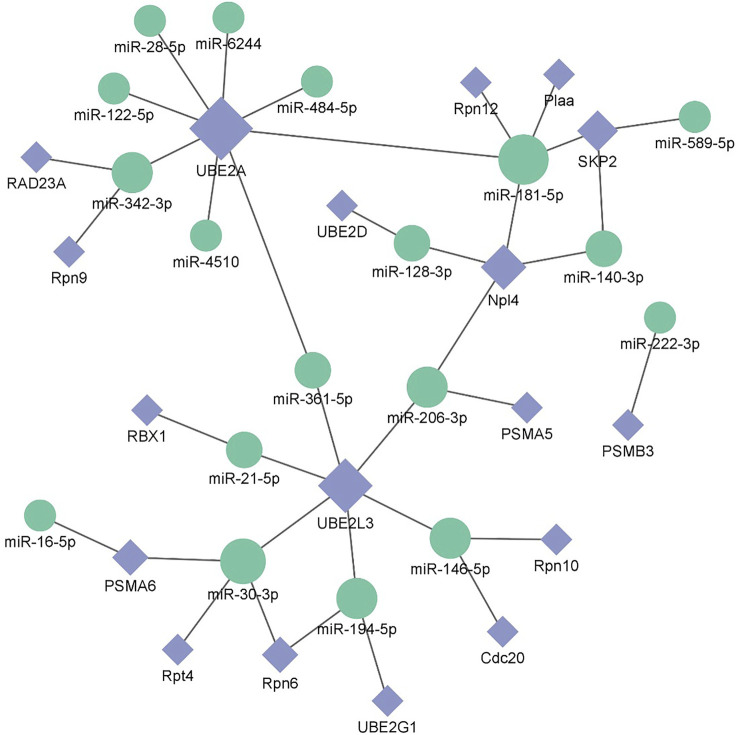
Potential miRNA-mRNA correlation network is related to the degradation of misfolded protein in ER.

After screening and excluding unidentified miRNAs and targeted putative proteins (proteins with unknown specific functions), 28 targeted miRNAs were obtained. According to the results of KEGG and GO analysis, 18 miRNAs targeting 18 mRNAs related to misfolded proteins in endoplasmic reticulum. Among 18 DEGs, UBE2D was the most significantly up-regulated gene (Figure 11). MiR-589-5p was the most down-regulated miRNA, followed by miR-30-3p (Figure 12). The expression of UBE2A, UBE2L3, and Npl4 was mainly regulated by miRNAs. We found that miR-181-5p and miR-30-3p regulated the most target genes, suggesting that miR-181-5p and miR-30-3p played a vital role in L. *vannamei* response to acute exposure to MC-LR (Figure 13).

## Discussion

In previous studies, acute toxicity studies of MC-LR have been conducted on some aquatic animals (Gonçlves-Soares et al., 2012; Hou et al., 2014; Chi et al., 2022). 48 h EC50 and 48 h LD_50_ of MC-LR to *Eriocheir sinensis*and and *Danio rerio* were 50 and 328 μg/kg BW, respectively (Hou et al., 2014; Chi et al., 2022). The mortality rate of *L. vannamei* was 100% after 96 h of inoculation with 200 mg/kg MCs, in which MC-LR was the most abundant MC (Gonçlves-Soares et al., 2012). In present study, 72 h LD_50_ of MC-LR to *L. vannamei* was 350 μg/kg BW. It was suggested that the tolerance of aquatic animals to MC-LR is species-specific, and differences in growth stage and culture environment will lead to different tolerance to MC-LR for aquatic animals of the same species.

In this study, a total of 1229 DEGs were obtained, including 844 up-regulated genes and 385 down-regulated genes. The oxidoreductase activity was significantly up-regulated in the GO molecular function category. Global and overview Maps (168), signal transduction (80), and endocrine system (67) were the most abundant pathways for enrichment of DEGs. To elaborate the toxicity mechanism of MC on fish and the detoxification mechanism of fish, silver carp were injected with 200 μg/kg MC-LR, GO analysis in RNA-Seq indicated that oxidoreductase activity was one of the significant subcategories in molecular function class. KEGG pathway analysis showed that DEGs were mainly concentrated in signal transduction, global and overview maps and endocrine system pathways (Qu et al., 2018).

To determine the active regulation function and pathway of miRNA in hepatopancreas of *L. vannamei* after MC-LR exposure, KEGG pathway analysis was performed on miRNA target genes. We found protein processing in ER was the most enriched pathway after MC-LR exposure. In eukaryotes, the 26S proteasome handled most regulated proteolysis and was pivotal for proper cell function. An important function of selective proteolysis was to remove misfolded proteins (Kim et al., 2006). ER protein processing and ER stress were observed in *β*-cell treated with MC-LR, and ER impairment was an important factor leading to MC-LR-incuced *β*-cell failure (Zhao Y. et al., 2017). After human hepatocytes were exposed to MC-LR, ER stress-related pathways and unfolded protein response (UPR) were highly enriched by DEGs (Biales et al., 2020). Based on these results, it was speculated that the pathway related to ER protein processing played an important role in the mechanism of MC-LR toxicity to hepatopancreas.

There is a strict protein quality control system in eukaryotic cells, and endoplasmic reticulum-associated protein degradation (ERAD) is an important part of this system. ERAD is responsible for reverse transport of misfolded and incompletely folded protein from ER to cytoplasm, where misfolded and incompletely folded protein are further degraded by 26S proteasome (Römisch, 2005; Kim et al., 2006). Only properly folded proteins are allowed to proceed to their destination to perform their physiological functions. In this study, the proteasome pathway (ko03050) was most enriched in hepatopancreas of *L. vannamei* after acute exposure to MC-LR, indicating that the degradation of misfolded proteins might be activated. ERAD played an important role in cell cycle regulation and signal transduction (Pickart, 2004). In addition, ERAD was also involved in tumorigenesis (Rajkumar et al., 2011). Our results showed that DEGs significantly enriched in the ERAD system after MC-LR infection.

Previous studies have found environmental stress could induce ER stress (Liang et al., 2016). However, most research mainly focused on unfolded protein response pathway (Chen et al., 2012; Yuan et al., 2017; Xu et al., 2018; Qu et al., 2021), whereas the response of ERAD to environmental stress was rarely reported. In this study, the analysis of KEGG pathway revealed that 18 genes were significantly up-regulated after MC-LR exposure for 72 h, among which Npl4 and RAD23A played an important role in substrate transfer from ER to proteasome. The function of Npl4 is to form p97-Ufd1-npl4 complex with p97 and ufd1, and then transfer protein on ER membrane to cytoplasm (Ye et al., 2001). The Pngl-Rad23 complex couples proteins to deglycosylation and degradation, and facilitates direct transfer of deglycosylated substrates to the proteasome (Kim et al., 2006). UBE2A, UBE2D, UBE2G1, and UBE2L3 belong to the UBE2 family. UBE2A binds to the ubiquitin ligase RAD18 to regulate the cell cycle and induce the process of multimeric ubiquitination (Ramatenki et al., 2015). RBX1 and SKP2 are components of Cullin-RING E3 ubiquitin ligase (CRL), and RBX1 is responsible for connecting with UBE2 and promoting the ubiquitination of protein substrate (Zhao and Sun, 2013). Rpn6, Rpn7, Rpn9, Rpn10, Rpn12, Rpt4, and Rpt5 are the components of 19S regulatory particle of proteasome. Rpn10 is one of ubiquitination receptors in proteasome, which is involved in the binding of polyubiquitination protein substrate (Wang et al., 2005). In addition, the expressions of PSMA5, PSMA6, and PSMB3 in 20S core particle of proteasome were significantly up-regulated. Therefore, it is speculated that MC-LR exposure may induce ERAD and UPP pathways and affect the degradation of misfolded protein in ER.

MiRNA plays an important role in biological regulation. In recent years, some studies have found miRNA could regulate the degradation of misfolded protein in ER (Wu and Pfeffer, 2016; Sengupta, 2017; Du et al., 2021). In our study, 18 miRNAs involved in misfolded protein degradation pathway in ER changed significantly after MC-LR exposure, and miR-181-5p was down-regulated. Previous studies have revealed that miR-181 family plays an important role in inducing necrotic apoptosis of cells (Cui et al., 2019), the occurrence and development of diseases (Su et al., 2014; Liang and Xu, 2020), body metabolism regulation (Abu-Farha et al., 2019; Wang et al., 2019). Moreover, miR-181a can regulate the immune response by affecting T cells (Bellon et al., 2009; Xie et al., 2013). After acute exposure to MC-LR, the expression levels of miR-181a-3p in the liver of juvenile silver carp (*Hypophthalmichthys molitrix*) was significantly decreased in 200 μg/kg groups at 1, 3, and 6 h, upregulated at 24 and 48 h, it may participate in the MC-LR-induced inflammatory response (Feng et al., 2020). After methamphetamine injection, the expression of miR-181a was down-regulated in rat brain, targeting genes involved in protein ubiquitination process and indirectly regulating the down-regulation of GABAAα1 expression (Wang et al., 2021). MC-LR prenatal exposure led to the impairment of learning and memory function in offspring on postnatal days 35, accompanied by ER stress and neuronal apoptosis in hippocampal CA1 regions of mice, and miR-181a-5p significantly downregulated (Liu et al., 2020). In human, miR-181a-5p may contribute to the development femoral head osteonecrosis after femur neck fracture *via* ubiquitin proteasome system (Chen et al., 2020). In this study, miR-181-5p regulated many genes of protein degradation pathway. We speculate that miR-181-5p might play an important role in the effect of MC-LR on the degradation pathway of misfolded protein, which requires further study.

In conclusion, this study provides a reference for studying miRNAs and mRNAs expression profiles in *L. vannamei* hepatopancreas after MC-LR exposure. These results are helpful to further study the mechanism of MC-LR toxicity to hepatopancreas of crustaceans. Further researches are required to verify miRNA-related target genes and explore their potential functions and molecular mechanisms.

## Data Availability

The raw data supporting the conclusions of this article will be made available by the authors, without undue reservation.
